# Identifying marine invasion threats and management priorities through introduction pathway analysis in a remote sub‐Antarctic ecosystem

**DOI:** 10.1002/ece3.11299

**Published:** 2024-04-23

**Authors:** Daniel T. I. Bayley, Paul E. Brewin, Ross James, Arlie H. McCarthy, Paul Brickle

**Affiliations:** ^1^ South Atlantic Environment Research Institute Stanley Falkland Islands; ^2^ Centre for Biodiversity and Environment Research University College London London UK; ^3^ Shallow Marine Surveys Group Stanley Falkland Islands; ^4^ Government of South Georgia & the South Sandwich Islands Stanley Falkland Islands; ^5^ Helmholtz Institute for Functional Marine Biodiversity at the University of Oldenburg (HIFMB) Oldenburg Germany; ^6^ Alfred‐Wegener‐Institut Helmholtz‐Zentrum für Polar‐ Und Meeresforschung Bremerhaven Germany; ^7^ School of Biological Sciences (Zoology) University of Aberdeen Aberdeen UK

**Keywords:** non‐native species, polar ecosystems, biosecurity, marine management, network analysis

## Abstract

The threat from novel marine species introductions is a global issue. When non‐native marine species are introduced to novel environments and become invasive, they can affect biodiversity, industry, ecosystem function, and both human and wildlife health. Isolated areas with sensitive or highly specialised endemic species can be particularly impacted. The global increase in the scope of tourism and other human activities, together with a rapidly changing climate, now put these remote ecosystems under threat. In this context, we analyse invasion pathways into South Georgia and the South Sandwich Islands (SGSSI) for marine non‐native species via vessel biofouling. The SGSSI archipelago has high biodiversity and endemism, and has historically been highly isolated from the South American mainland. The islands sit just below the Polar Front temperature boundary, affording some protection against introductions. However, the region is now warming and SGSSI increasingly acts as a gateway port for vessel traffic into the wider Antarctic, amplifying invasion likelihood. We use remote Automatic Identification System vessel‐tracking data over a 2‐year period to map vessel movement and behaviour around South Georgia, and across the ‘Scotia Sea’, ‘Magellanic’ and northern ‘Continental High Antarctic’ ecoregions. We find multiple vessel types from locations across the globe frequently now enter shallow inshore waters and stop for prolonged periods (weeks/months) at anchor. Vessels are active throughout the year and stop at multiple port hubs, frequently crossing international waters and ecoregions. Management recommendations to reduce marine invasion likelihood within SGSSI include initiating benthic and hull monitoring at the identified activity/dispersion hubs of King Edward Point, Bay of Isles, Gold Harbour, St Andrews Bay and Stromness Bay. More broadly, regional collaboration and coordination is necessary at neighbouring international ports. Here vessels need increased pre‐ and post‐arrival biosecurity assessment following set protocols, and improved monitoring of hulls for biofouling to pre‐emptively mitigate this threat.

## Graphical abstract

South Georgia and the South Sandwich Islands (SGSSI) currently has only one documented established non‐native species. However, the combination of a rapidly changing climate and an increase in tourism activities make these previously isolated islands now at risk, and in need of evidence‐based knowledge of key locations and vectors for assessing potential non‐native species spread. In our paper we analyse the threat to SGSSI from marine Invasive non‐native species using Automatic Identification System (AIS) remote vessel‐tracking data. We use a combination of network analysis and vessel movement analysis of behaviour and characteristics to assess ‘invasion pathways’, and to identify threats across three South Atlantic ecoregions. This work aims to guide pre‐emptive management and effective long‐term monitoring.

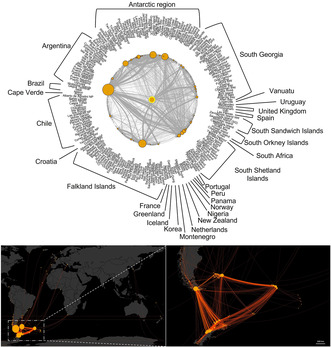

## 1 INTRODUCTION

Marine invasive species can threaten biodiversity, industry, and both human and wildlife health (Bax et al., 2003). Invasive species can also cause significant damage to ecosystems through habitat disturbance, competition, predation, induced toxicity and genetic introgressive hybridisation. In extreme cases, loss of ecosystem function, extinctions or structural change of whole landscapes can occur (Jeschke et al., 2014; Ricciardi & Cohen, 2007; Simberloff, 2011). The process leading to these environmental impacts begins with the introduction and establishment of species in an area beyond their native ranges (Blackburn et al., 2011; Jeschke et al., 2014). Once a species has become established, subsequent control and remediation measures can be both difficult and costly for ecosystems as well as infrastructure (Marbuah et al., 2014). The threat from marine invasive species is a global issue, where <16% of marine ecoregions have no reported invasions (Molnar et al., 2008), and new global primary detections of aquatic non‐indigenous species have occurred at a rate of roughly one new detection every 8.4 days for 50 years (Bailey et al., 2020). Moreover, there is often no data available to establish baselines and monitor for coastal introductions, particularly in remote locations (Varnham, 2006), meaning real introduction numbers may be higher still.

Species that arrive in new locations by anthropogenic means are considered non‐native, regardless of their level of impact (Lockwood et al., 2013), yet each new introduction has the potential to become invasive. Precautionary management includes pathway‐focused practices that prevent or minimise the introduction of any non‐native species via major dispersal vectors including ballast water release, biofouling of hulls and internal seawater systems, and equipment contamination (Bailey et al., 2020; Bax et al., 2003; Davidson et al., 2021; Molnar et al., 2008). The Antarctic and sub‐Antarctic regions are some of the most remote and inaccessible locations on Earth, and were once thought to be essentially impenetrable to marine non‐native species due to the remoteness and extreme environments. Now, however, this region's climate and accessibility is rapidly changing (Chown et al., 2012; Clarke et al., 2005; Hughes et al., 2020; McCarthy et al., 2019, 2022). New introductions and successful establishments within the sub‐Antarctic are considered more likely in lower‐latitude areas that are warmer and closer to a mainland, such as the archipelago of South Georgia (Chown et al., 2012; Hughes et al., 2020).

Non‐native species dispersal through ballast water (Dulière et al., 2022; Lewis et al., 2003; McCarthy et al., 2019) is globally regulated (though not strictly implemented) through the IMO Ballast Water Management Convention (IMO, 2004), and recommendations specifically for the polar regions are outlined in the Antarctic Ballast Water Guidelines (IMO, 2007). These guidelines require exchange or release of ballast waters offshore (north of either the Polar Frontal Zone or 60° S, and at least 200 nautical miles from the nearest land). Regular maintenance is mandatory, alongside log‐keeping, and internal mitigation treatment. However, the other major introduction pathways of biofouling (on hulls and within internal seawater systems), are still largely unmitigated, aside from broad guidance such as the IMO Biofouling Guidelines (International Maritime Organisation, 2023). This represents a significant, unmanaged threat to marine biodiversity (Bax et al., 2001).

The Scotia Sea ecoregion (Spalding et al., 2007) is made up of South Georgia and the South Sandwich Islands (SGSSI), the Antarctic Peninsula, South Orkney and the South Shetland Islands. This ecoregion has few historical recordings of non‐native species and these are almost entirely terrestrial non‐native species (Frenot et al., 2005). However, multiple non‐native marine algae and invertebrates have been observed within the nearby Antarctic Peninsula region (Cárdenas et al., 2020; McCarthy et al., 2019), and the first record of an established marine non‐native (*Ulva fenestrata*) within South Georgia waters was recently recorded (Mrowicki & Brodie, 2023). Despite low‐level passive dispersal of marine non‐native species (Avila et al., 2020; Brasier et al., 2021), increasingly frequent rafting on kelp or plastic transports species to the archipelago (Convey & Peck, 2019; Fraser et al., 2018; Griffiths & Waller, 2016). Nonetheless, most current introductions to this region are more likely facilitated via vessel biofouling or through poorly maintained, emergency or illegal vessel ballast release (McCarthy et al., 2022). Despite this, exact routes, frequency and composition of vessel traffic into this ecoregion are poorly understood, and especially which vessels' movement behaviours are more likely to introduce non‐native species.

SGSSI's location just south of the Polar Frontal Zone and north of the Antarctic Circumpolar Current Front, means it acts as both a Northern and Southern range limit for many species (Griffiths et al., 2009; Hogg et al., 2011; Queirós et al., 2024). This biogeographic isolation and the increasing number of international vessels frequently crossing the natural barrier of the fronts, makes this area at growing risk of invasion (Hughes et al., 2020; Kennicutt et al., 2019; McCarthy et al., 2019). A recent global analysis by McCarthy et al. (2022) of ship traffic travelling into the neighbouring Antarctic further found the Scotia region to have the greatest and most diverse volume of traffic passing through their port hubs, making SGSSI a key ‘gateway port’ location.

Here we analyse potential for marine non‐native species to be introduced via ships to SGSSI through analysis of AIS (Automatic Identification System) vessel tracking. We conduct a regional‐scale network analysis and spatial assessment of vessel movement across the South American sub‐Antarctic (across an area of ~8.5 million km^2^) to assess these potential marine introduction routes. To inform invasion mitigation and planning for this remote archipelago, we highlight major factors associated with vessel movement and behaviour that increase the potential for introductions. Finally, we set out potential biosecurity controls for inshore vessel management, and list priority sites for monitoring. These management actions aim to help protect the unique biodiversity of SGSSI's marine and coastal ecosystems. Pre‐emptive management here and in neighbouring major ports to reduce invasion likelihood will be essential for safeguarding the biodiversity of the wider Antarctic and sub‐Antarctic wilderness.

## 2 MATERIALS AND METHODS

This study focuses on vessel movement patterns from traffic in and out of the UK Overseas Territory (UKOT) of SGSSI, and their connecting stops before and after arrival. Vessel location data were recorded remotely within an area of interest (AOI) that included SGSSI's ecoregion of the ‘Scotia Sea’, and the surrounding ecoregions of ‘Magellanic’ and the northern ‘Continental High Antarctic’.

South Georgia and the neighbouring South Sandwich Islands are relatively isolated geographically, and a large (1.24 million km^2^) IUCN category VI Marine Protected Area has protected SGSSI administratively since 2012 (UNEP‐WCMC, 2021). There is no permanent population on South Georgia or the smaller islands, and there is an average annual presence of ~40 people. Small settlements are located in Grytviken and King Edward Point (on South Georgia), and on neighbouring Bird Island.

Automatic Identification System tracking data were assessed over a 2‐year period (running 1 July 2017 to 30 June 2019), from austral winter to austral winter, at an hourly resolution. Hourly resolution was chosen to limit the number of position reports, while maintaining critical movement and behavioural information. The data therefore detail the path travelled by each vessel when underway (speed >0.2 knots) and any stop locations (≤0.2 knots or moving <400 m over 1 h), following standard *transit simplification* data cleaning recommendations from MMO (2013).

**FIGURE 1:**
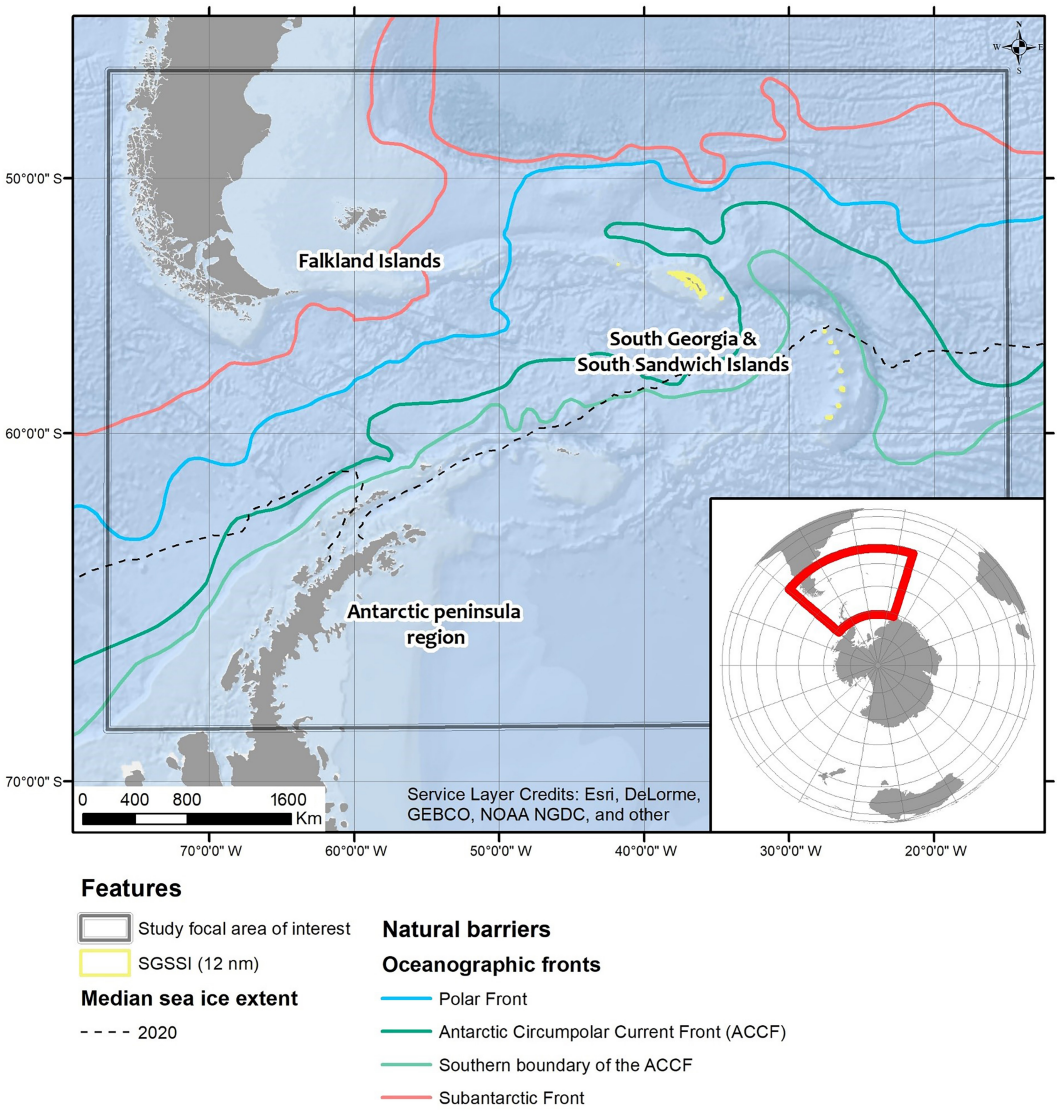
Map detailing the study area of interest for analysis of vessel movement in and out of South Georgia and the South Sandwich Islands' waters. Key features: Study focal area (containing Automated Identification System (AIS) data for period July 2017–2019), winter sea‐ice extent (2020) and major regional oceanographic current fronts.

A 2‐year period ensured any anomalies associated with any particular year were accounted for. Results are mean averages over the 2 years. These data were analysed for all vessels with AIS transmissions, within a defined AOI, ranging from 68.5° S to 45° S latitude, and 77° W to 15° W longitude (Figure 1). International Maritime Organisation (IMO) regulations requires AIS to be fitted onboard: (1) all ships of 300 gross tonnage and upwards engaged on international voyages, (2) cargo ships of 500 gross tonnage and upwards not engaged on international voyages and (3) all passenger ships irrespective of size (International Maritime Organisation, 2015). AIS compliance by vessels is considered very high for SGSSI, and matches the mandatory permit records required for all vessels entering SGSSI waters.

Data attributes used in the analysis included vessel Maritime Mobile Service Identity (MMSI) number, IMO number and vessel name. Movement details included: Time stamp (UTC date and time), Latitude and Longitude (WGS84, DGPS, Loran‐C), Course (degrees), Status (e.g. moored, underway), Speed in knots and (vessel‐specified) major port of origin. Vessel dimension data included: Length overall (L_OA_) in m, Breadth overall (B_OA_) in m, Volume/Gross Tonnage (V), Dead Weight Tonnage (DWT), Draft (T) in m and Vessel type (Bulk, Cargo, Fishing, Offshore, Passenger, Pilot, Pleasure, Tanker and Tender). Data were supplied by ‘BigOceanData’ (https://www.bigoceandata.com).

### 2.1 Data cleaning and node definition

Data were initially cleaned and filtered to remove any points associated with vessels (classed by unique MMSI codes) which never entered within the SGSSI Maritime Zone (200 nm limit) during the study period. ‘Nodes’ (spatial clustering of vessel activity signifying ports or temporary anchorages) were created following Letschert et al. (2021). With the Geosphere package (Haversine function) in R (version 4.1.1/RStudio v1.4.1717) we calculated the sequential AIS point‐to‐point distances for each vessel within the AOI, from hourly AIS signal data over time. These sequential point data were then filtered to only include stationary vessels (i.e. anchored, moored, at port or using Dynamic Positioning), which likely present higher propagule release in that location. Stationary vessels were defined as those moving <400 m over 1 h (equivalent to a speed of ~0.2 knots, as per MMO (2013) guidance), and with an AIS ‘Speed’ classification of <1 knot. The combination of these two variables ensured that the analysis only included vessels at anchor or stationary holding position, and accounted for any potential signal errors in either one of the location or speed attributes.

We created network nodes for all clusters of stationary vessels within 12 nm of land. All buffers were created using a World Azimuthal Equidistant projection. We created network nodes using a 5‐km buffer, based on clustering of stationary vessels, and linked these locations to closest major ports, known anchorage or geographical features. Buffers were spatially joined to vessel stop points to link location to event. Unique vessel ‘events’ were calculated based on cumulative time stopped at a location for each vessel (over a unique continuous period of time). We limited the analysis to prolonged stationary periods rather than all passing traffic, as longer periods at port are known to increase the opportunities for organisms to both attach to hull surfaces and for them to spread into the new environment (Sylvester et al., 2011). In addition to nodes created inside the AOI, if vessel‐specified ‘port of origin’ data were different to the known origin node identified through AIS point analysis (i.e. outside the AOI), we used this data to identify broader global port links. This amalgamation of data types gives a clearer picture of pathways into the region. There may, however, have been additional intermediate stops in between the stated origin port and the time the vessel enters our AOI. All locations in the study were also assigned to a recognised country or territory in order to group regional activity.

### 2.2 Network analysis and route (edge) definition

Networks were created using the igraph package in R (Csardi & Nepusz, 2006) to visualise vessel route linkages (edges) between all vessel anchorages (nodes) and compute the frequency of journeys to and between them by vessels. Network node size was based on the total number of visits during the study period. Edge connection routes and ‘weight’ were calculated based on the frequency of unique vessel trips along each port‐to‐port route. The factors included here (Table 1), such as number of ports/regions visited, and period of time in transit (i.e. without hull cleaning) are known key factors increasing colonisation pressure (i.e. invasion potential) from accumulated new species (Davidson et al., 2016; McCarthy et al., 2019; Sylvester et al., 2011). The hull condition (i.e. frequency of cleaning and therefore level of biofouling) is not known for any vessel in this analysis. While factors including hull condition, biofouling species composition, and environment characteristics of start and end port are important considerations affecting colonisation pressure we had to limit this analysis to factors based on vessel design and movement behaviour. Length of time travelling without prolonged periods at rest in port is therefore used as an indicative proxy of this unknown biofouling extent element.

**TABLE 1:** Study factors known to increase the likelihood of non‐native species introduction, spread, settlement and establishment.

Locations	Higher values infer:
Total number of visits	↑ Likelihood of initial introductions
Overall WSA within port	↑ Hull substrate area for biofouling and transport
Period of time stopped at anchor	↑ Likelihood of settlement, establishment and dispersal
Number of identified links to port	↑ Likelihood of introduction and dispersal
Number of identified links from port	↑ Likelihood of dispersal
Number of vessel types using port	↑ Likelihood of introduction

Vessels	Higher values infer:
Total vessel number present	↑ Likelihood of initial introductions
Total WSA (m^2^)	↑ Hull substrate for biofouling and transport
Maximum number of extended ‘journeys’	↑ Likelihood of biofouling and dispersal. ↓Likelihood of hull cleaning
Maximum number of stops during extended ‘journey’	↑ Likelihood of introduction and dispersal
Total months of activity	↑ Likelihood of biofouling, dispersal and survival over more months ↓Likelihood of hull cleaning
Average stop time at port (hours)—within study area	↑ Likelihood of settlement, establishment and dispersal
Total number of ‘countries of origin’	↑ Likelihood of novel introduction and dispersal
Maximum number of trans‐national trips	↑ Likelihood of novel introduction and dispersal

*Note*: Factors split between location (i.e. applied to certain ports/anchorages) and vessels (i.e. applied to all vessels, split by type). Values calculated for annual periods and applied solely to the study area of interest. Refer McCarthy et al. (2019) and Davidson et al. (2016) for more details on types and mechanisms of known high‐risk factors.

### 2.3 Wetted surface area analysis

Wetted Surface Area (WSA) represents the potential of a vessel's hull to transport marine species which settle over time (Moser et al., 2016). WSA was calculated for each vessel (unique MMSI) following the method by Moser et al. (2016), using the Denny‐Mumford WSA regression formula, and grouping vessels using the nine standard classes (Bulk, Cargo, Fishing, Offshore, Passenger, Pilot, Pleasure (Yachts), Tanker and Tender). The ‘Pleasure’ vessel category incorporates both yachts and small motorised crafts (ranging from 14 to 69 min our study). As Moser et al. (2016) did not include small yachts/pleasure crafts, WSA calculations for pleasure vessels <26 m L_OA_ followed Bakker and van Vlaardingen (2017); Denny‐Mumford formula (WSA = 1.7 · LOA · T + V/T). Larger pleasure vessels used values from the ‘fishing vessel’ category from Moser et al. (2016). ‘Service’ vessels were split into ‘Pilot’ or ‘Tender’ vessel types. ‘Offshore’ vessels were entirely composed of research vessels and followed the ‘Other’ category from Moser et al. (2016).

WSA calculations used the equation *WSA = a DWT* ^*b*^, with ‘a’ = regression coefficient, ‘b’ = regression exponent and ‘DWT’ = Dead Weight Tonnage (Moser et al., 2016). If DWT values were unavailable for ‘Fishing vessels’, ‘Tugs and supply’ and ‘Passenger ships’, vessel ‘Breadth Overall’ (B_OA_) was used (with corresponding regression values). For ‘Other ships’, vessel ‘Length Overall’ (L_OA_) was used (with corresponding regression values). All individual tenders were classed as having 9.9 m^2^ WSA, based on Bakker and van Vlaardingen (2017) values for vessels 4–6 m in length.

### 2.4 Relative threat from different vessel types

This analysis assumes equal levels of hull maintenance and condition for all vessels, as monitoring and assessment is not currently underway. Our analysis, therefore, assumes that all vessel hulls have some (uniform) degree of biofouling, and each vessel has the potential to spread non‐native species propagules based on the size of that vessel and its behaviour alone. All vessels are assumed to comply with ballast water exchange regulation (outside of South Georgia waters). As such potential propagule release from ballast water is not considered here. By including WSA, this analysis explicitly differentiates biofouling on different vessel classes only in relation to hull fouling. While larger vessels typically have more extensive internal seawater systems and niche areas, the wetted area within such systems has not explicitly been calculated or included in this study.

## 3 RESULTS

### 3.1 Vessel analysis

**FIGURE 2:**
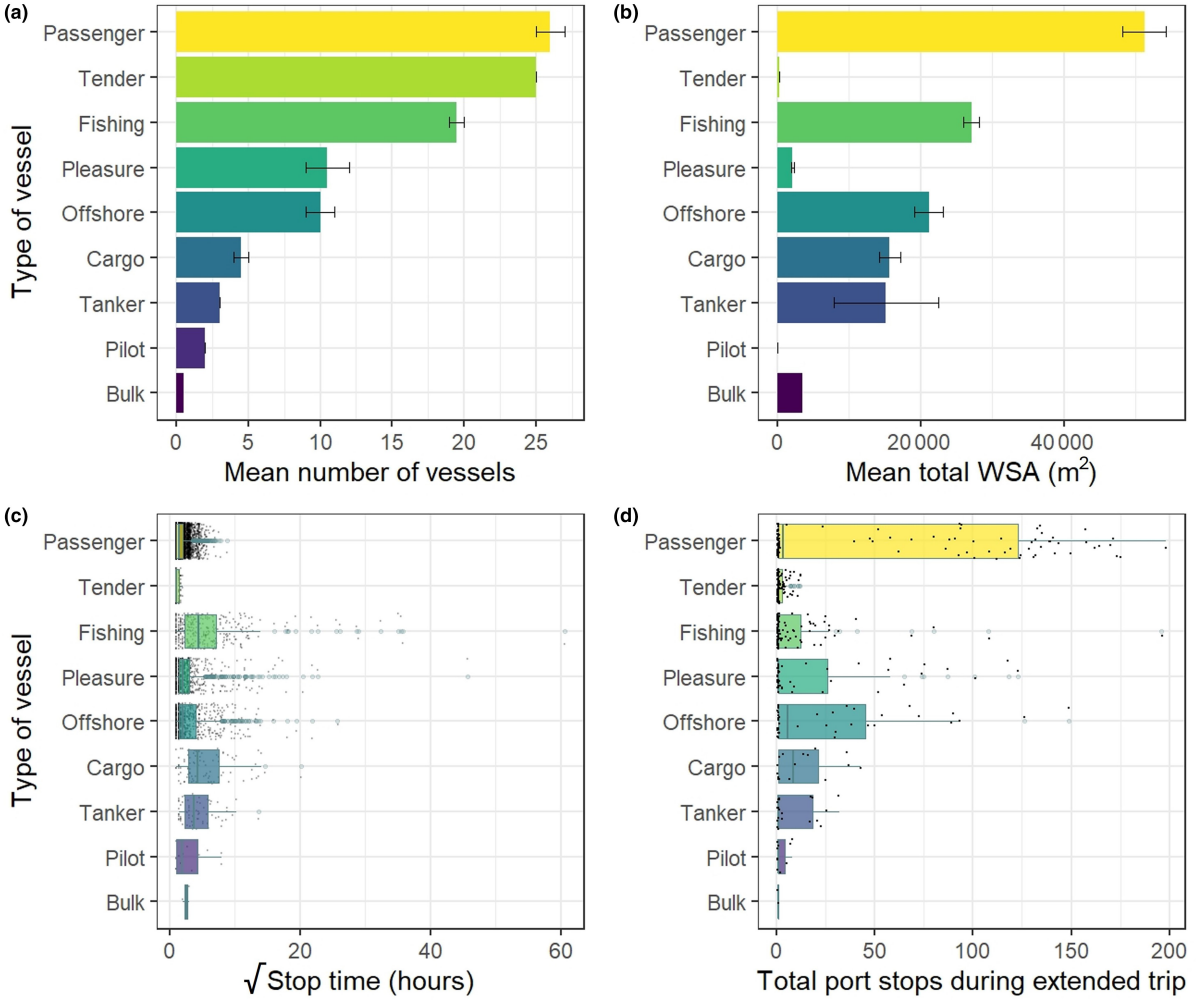
Vessel movement characteristics by vessel type, for all vessels entering SGSSI between July 2017 and 2019. Panels a and b show: Barplots of mean number of vessels active around SGSSI; and their cumulative Wetted Surface Area (WSA), with error bars ± SE. Panels c and d show: Boxplots of (square rooted) total stop time (hours) and number of separate locations stopped during a continuous extended trip (i.e. before a rest of period of >1 month). Boxplots show median and IQR (whiskers show max value in IQR, plus outlier points), with data points jittered over. All panels ordered by total number of vessels.

In total, 143 vessels entered the SGSSI maritime zone over the study period. Of these vessels, 123 (86%) stopped within 12 nm of land (noting that the total falls to 78 vessels when excluding vessel tenders). An average total of 100 separate vessels were present within any 1 year. Passenger and fishing vessels (and associated small tenders) were the most common vessel types entering within SGSSI Maritime Zone, with more passenger vessels than bulk, tanker, pilot and cargo vessels combined (Figure 2, Table 2). Similarly, cumulative WSA was largest for the passenger vessels. These vessels were followed by the mid‐sized vessels used for ‘fishing’ and ‘offshore’ research surveys. Despite their low frequency of occurrence, cargo and tanker vessels had a relatively high WSA due to their considerable size.

**TABLE 2:** Heat range map (red = highest, white = lowest) of annual mean vessel movement, and design characteristics associated with introduction of non‐native species within South Georgia.

Annual values by vessel type	Bulk		Cargo		Fishing		Offshore		Passenger		Pilot		Pleasure (yachts)		Tanker		Tender	
	Mean	SE	Mean	SE	Mean	SE	Mean	SE	Mean	SE	Mean	SE	Mean	SE	Mean	SE	Mean	SE
Vessel number	**0.5**	(0–1)	**4.5**	(4.1–4.9)	**19.5**	(19.3–19.7)	**10**	(9.8–10.2)	**26**	(25.9–26.1)	**2**	(2–2)	**10.5**	(10.2–10.8)	**3**	(2.4–3.6)	**25**	(24.8–25.2)
Cumulative WSA (m^2^)	**3429**	(0–6858)	**15678**	(15392–15964)	**27084**	(26767–27401)	**21152**	(20908–21397)	**51259**	(50997–51522)	**47**	(28–65)	**2126**	(2045–2208)	**15174**	(9968–20379)	**248**	(248–248)
Number of extended ‘journeys’	**2**	(0–4)	**1.8**	(1.3–2.2)	**2.3**	(2–2.7)	**1.9**	(1.6–2.2)	**2**	(1.9–2)	**2.5**	(0.8–4.2)	**2.3**	(2–2.7)	**3.3**	(1.6–5)	**2**	(1.8–2.1)
Number of stops during extended ‘journey’	**1**	(1–1)	**13.6**	(6.2–20.9)	**11.4**	(6–16.8)	**29.4**	(16.8–42)	**57.5**	(44.8–70.2)	**2.8**	(1–4.6)	**20.6**	(10.9–30.2)	**8.6**	(3.9–13.3)	**2.5**	(2–3)
Stop time at port (hours)—within study area	**6.5**	(1.5–11.5)	**45.1**	(31.8–58.5)	**71.4**	(45–97.7)	**19.4**	(15.5–23.2)	**4**	(3.9–4.2)	**15.3**	(3.3–27.2)	**19.7**	(12.7–26.8)	**24.6**	(17.3–32)	**1.4**	(1.3–1.5)
Total number of ‘countries of origin’	**1**	(1–1)	**7**	(6–8)	**12**	(9–15)	**12**	(10–14)	**11**	(4–18)	**5**	(5–5)	**11**	(8–14)	**8**	(7–9)	**14**	(10–18)
Number of trans‐national trips	**0**	(0–0)	**5.7**	(0.3–11)	**3.2**	(2.1–4.2)	**10.5**	(6–15)	35	(32.1–37.9)	**0.8**	(–0.8–2.3)	**7.9**	(5.6–10.2)	**2.7**	(0.2–5.1)	**1.5**	(0.9–2)

*Note*: Analysis is split by vessel type. All values are based on AIS data assessed over the period 2017–2019. The items in bold represent the main value of interest (the Mean), while the SE value just illustrates the error around those mean values.

Vessels typically made a small number of extended journeys (i.e. consecutive multi‐stop journeys with no prolonged intervening stop period) during each year within the AOI (Figure 2, Table 2). Each of these journeys was followed by long periods (>1 month) stopped at port. Tankers had the highest average number of extended journeys within a year (mean = 3.3, *n* = 6, range = 2–6), and cargo vessels the lowest (mean = 1.8, *n* = 9, range = 1–3), further data summaries are shown in Appendix S1. Extended periods of inactivity were dominated by fishing and pleasure vessels (Figure 2), with some individuals of these vessel types inactive for >4 months. Offshore survey vessels and cargo freighters likewise stayed stationary for multiple weeks. Mean stationary time at port ranged from 71.4 h (*n* = 354, range = 1–3675 h) for fishing boats to 1.36 h (*n* = 215, range = 1–4 h) for tenders. Total port stops during extended journeys were highest for the passenger vessels, which had a mean average of 57.5 stops (*n* = 102, range = 1–198), and lowest for the bulk carrier with a mean average of 1 stop (*n* = 2).

**FIGURE 3:**
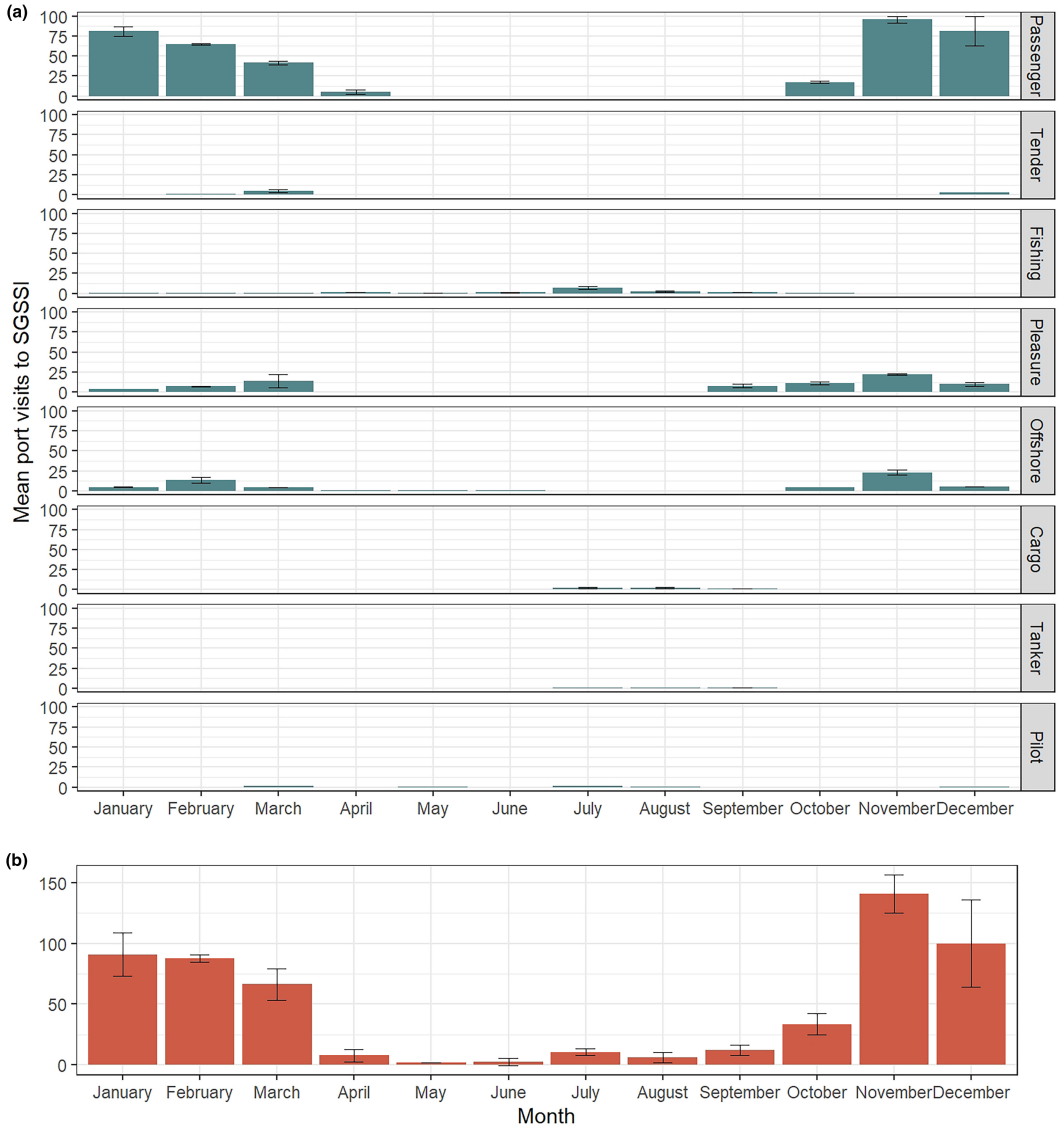
Mean number of visits by month for all vessel types present within SGSSI over the year. Data averaged across 2 years (2017–2019). Panel a = split by vessel type, panel b = all vessels. Error bars detail mean ± SE.

Vessels were present throughout the year. However, vessels start arriving inshore in abundance from October (Figure 2), corresponding to the tourist season and changes in animal activity (e.g. the arrival of penguins), reduced ice cover and increased daylight. November was the peak period with a mean of 126 unique visits over the 2 years, and mean visits each month afterwards ranged from 90 to 56 per month until April. Low activity season ranges from April to September and was at a minimum in May/June with two to three unique visits per month (Figure 3).

Breaking this activity down into vessel type, passenger vessels were most abundant (followed by pleasure yachts), and generally occurred October to March during the warmer Summer/Autumn months. Fishing vessels, while less numerous, occurred throughout the year (except summer). Similarly, research vessels occurred in all months except Spring. Tankers and cargo vessels were highly seasonal, occurring only in Spring during the analysis period (Figure 3).

#### 3.1.1 Locations

**FIGURE 4:**
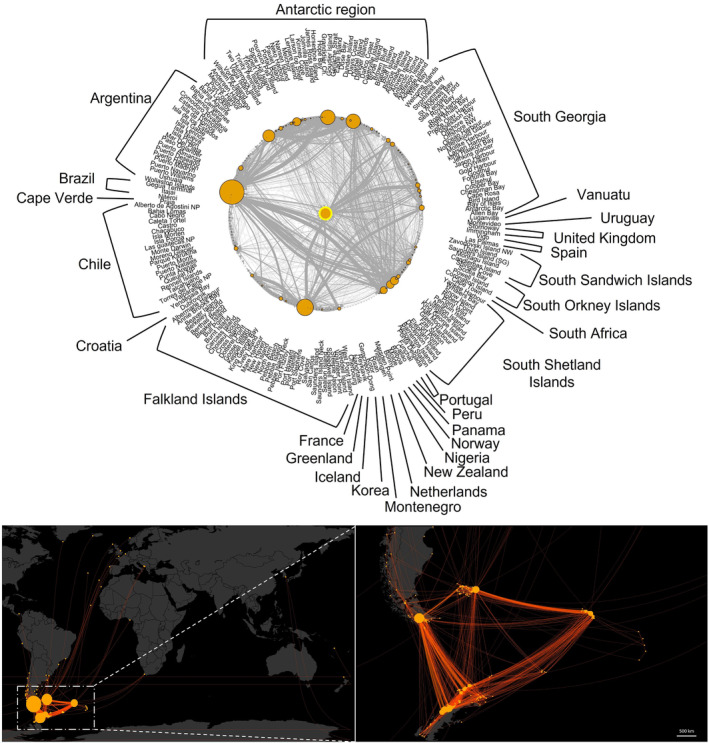
Countries of origin and number of vessels entering the South Georgia and South Sandwich Islands during the period 2017–2019. Within all panels, larger node (orange circles) size and edge (grey/orange lines) thickness indicates higher vessel frequency, based on known movements, averaged over 2 years. Analysis includes a total of 28 countries of origin (inclusive of the shared Antarctic Peninsula region). Top panel centre node (highlighted with a yellow ring) is King Edward Point, South Georgia. Bottom panels show idealised direct routes from port to port for each vessel within the analysis.

Vessels originated from 29 countries/territories, with South Georgia counted separately from the South Sandwich Islands (Figure 4). Most of the journeys into South Georgia started in the Falkland Islands and the Antarctic Peninsula. For most of the vessels, the first observation in our data (i.e. their initial known port or anchorage in their unique journey according to AIS transmission or records) was in South Georgia itself. It is important to note here however that AIS data shows that all these vessels (aside from the pilot vessels) do typically leave South Georgia waters and return to the region at least once annually. All vessels do therefore pose a threat of introducing new species to the region and dispersing them locally or regionally. Other vessels (from individual vessels to >60 from a single country), came from locations across Europe, Africa, the Pacific, Asia, central and South America, and the Arctic, primarily from South America. Most vessels entering directly into South Georgia (i.e. based on location of the last port of call before South Georgia) were from the Falkland Islands, followed by boats moving around from location to location within South Georgia, as well as vessels from within the Scotia Arc region, Patagonia and the Antarctic.

**FIGURE 5:**
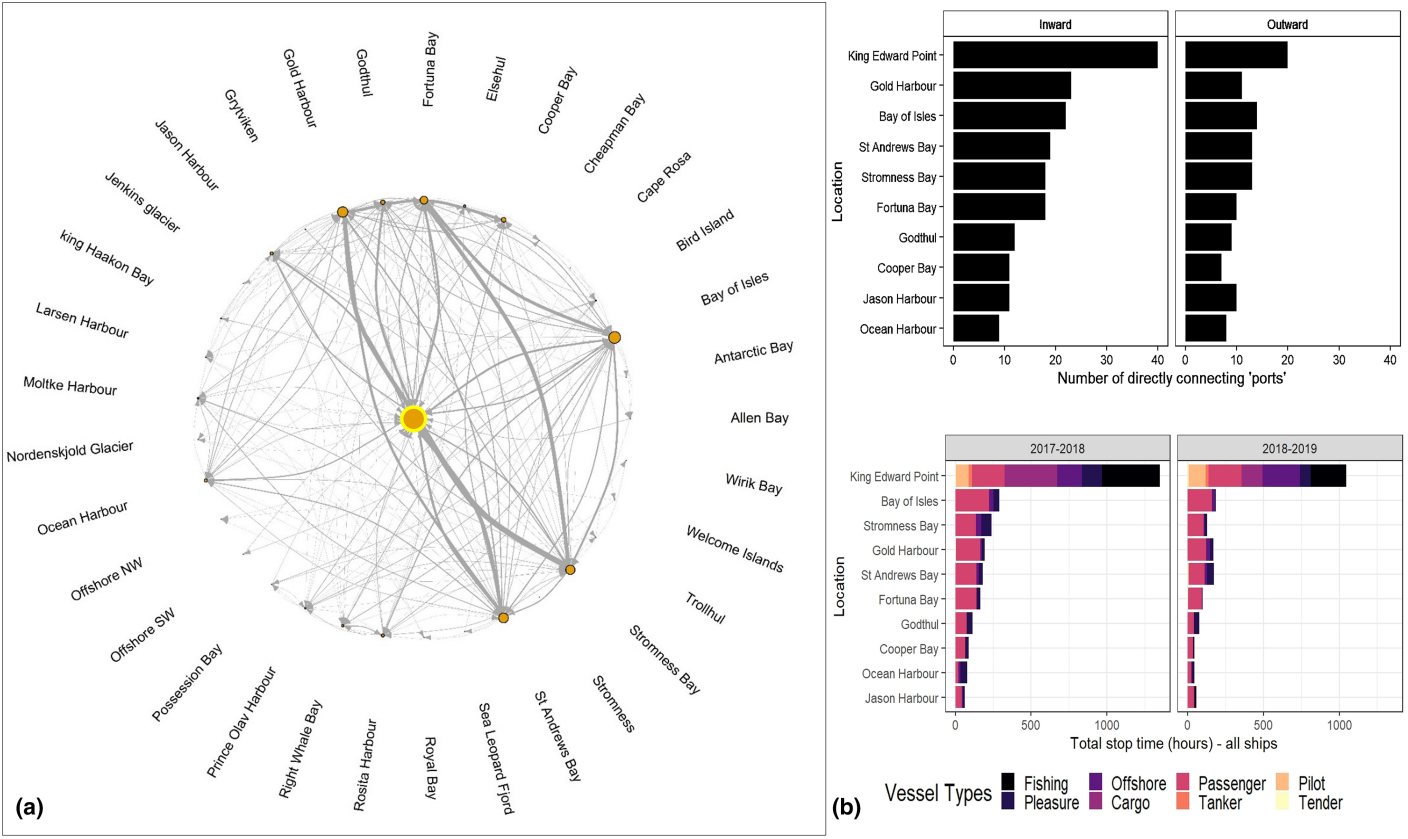
Local SGSSI vessel characteristics: (a) Network analysis of local vessel routes, route frequency (edges) in grey and abundance of vessels (nodes) in orange at each local anchoring location within South Georgia (central orange node with yellow ring indicates King Edward Point). Data averaged across 2 years from 2017 to 2019; (b) Number of immediately connected port locations in and out of the 10 busiest South Georgia locations, and vessel activity at each of these 10 locations within South Georgia.

The top 10 locations for vessel activity within SGSSI over the study period (Figure 5) were identified: King Edward Point (KEP)/King Edward Cove had the highest number of vessels and connections, followed by Bay of Isles, Gold Harbour, St Andrews Bay and Stromness Bay. As expected, KEP had the most inward links, with 40 linked mooring sites/ports, followed by Gold Harbour with 23. KEP has a large number of separate visits throughout the year, by all ship types, and with vessels averaging 10 h stationary (up to a maximum of 159 h within the study period). KEP therefore receives a high WSA of hulls from diverse origins in the water over a relatively prolonged period.

The site with most outward links was again KEP with 20 linked sites, followed by Bay of Isles with 14. The sites with the greatest number of *initial* entry/first port‐of‐call stops into South Georgia from other countries across the region were primarily KEP, Gold Harbour, St Andrews Bay, Cooper Bay, Bay of Isles and Stromness Bay.

#### 3.1.2 Vessel behaviour and specification factors

Passenger vessels appear to present the highest overall threat of the vessel types entering South Georgia (Table 2, Appendix S1). Passenger vessels were the most abundant (26, CI = 25.9–26.1) of vessels annually entering SSGGI waters, and all with relatively high WSA, resulting in the largest cumulative WSA annually (51,259 m^2^, CI = 50,997–51,522) by a large margin. Passenger vessels also had the highest average number of stops in different locations during their voyages (57.5, CI = 44.8–70.2) were typically active 7 months of the year and crossed international waters with an average of 35 (CI = 32.1–37.9) times between 12 different countries or territories. Passenger vessels were followed by fishing vessels, offshore survey vessels and yachts. These were again characterised by large vessel numbers of intermediate size, crossing between multiple countries or territories on extended journeys throughout most of the year. Tankers, pilot and cargo vessels are considered of medium threat (Tankers and cargo vessels being very large, crossing multiple countries and stopping for extended periods). Bulk carriers are a lower threat due to their low occurrence within SGSSI waters. Tenders are a medium threat, but these are strongly linked to a variety of mother ship vessel types and are likely to be out of water often. KEP was the port around South Georgia with the highest likelihood of introductions across all factors. Ports with a medium likelihood of introductions were Bay of isles, Gold Harbour, St Andrews Bay and Stromness Bay (each with broadly equal likelihood of introductions).

#### 3.1.3 Species of concern

There are currently 12 identified species of concern for South Georgia, and 11 for the neighbouring Falkland Islands (Table 3), based on a Horizon Scanning workshop conducted in 2018 with regional experts (Roy et al., 2019). The marine species identified are primarily fully marine invertebrate filter‐feeders/omnivores, along with two types of algae and one saltmarsh grass (*Spartina* spp. excluded from this study as not spread via hull biofouling). Species' native ranges (original point of origin) are from across both the North and South Atlantic, Mediterranean, West Pacific and sub‐Antarctic regions (Hughes et al., 2020; Roy et al., 2019). All species are primarily epibenthic when adult, and are typically annual breeders with an extended larval phase, and high fecundity, originating from high Boreal/Austral or temperate marine environments. Many also demonstrate a tolerance to surviving a broad range of temperature and salinity levels (although less is known of their sustained reproductive capacity across these conditions). It is important to note that none of these species have yet been recorded in SGSSI and so there is a large amount of uncertainty regarding their real‐world survival and reproduction potential, and pivotally, whether other species, not considered here, will arrive first (e.g. Mrowicki & Brodie, 2023).

**TABLE 3:** Non‐native species with high likelihood of arrival, establishment and impacts within the Falkland Islands and South Georgia and South Sandwich Islands.

Species (ranked)	Common name	Priority threat^a^		Depth range (m)/Max known depth	Feeding method	Substrate (Soft/Hard/Biological)	Non‐larval mobility type	Life Span (years)	Maximum planktonic phase (days)	Temp range (˚C)	Salinity range (PSU)	Reproduction	Larval pelagic development	Potential Routes	References	Ref #2
		SGSSI	FI													
*Mytilus chilensis* ^b^	Chilean mussel	◯	◯	<20	Filter/suspension	S/H/B	Sessile/Crawler	Up to 24 (typically <3)	180	−1.8 to 29	10 to 35	Sexual (ext)	Planktotrophic	Hull	Degen and Faulwetter (2019)	
*Mytilus edulis*	Blue mussel	◯	◯	<20	Filter/suspension	S/H/B	Sessile/Crawler	Up to 24 (typically <3)	180	−1.8 to 29	10 to 35	Sexual (ext)	Planktotrophic	Hull	Degen and Faulwetter (2019)	https://invasions.si.edu/nemesis
*Undaria pinnatifida*	Asian kelp		◯	5–25	Photosynthetic	H/B	Sessile	1	14	0 to 27	20 to 37	Sexual (ext)/sporogenesis/vegetative	Gametophytic plankton	Hull	https://invasions.si.edu/nemesis	
*Botryllus schlosseri*	Colonial ascidian	◯	◯	<200	Active suspension feeder	H/B	Sessile	<1	2	−1 to 30	14 to 44	Sexual (ext)	Lecithotrophic	Hull	https://www.marlin.ac.uk/	https://invasions.si.edu/nemesis
*Carcinus maenas*	European shore crab	◯	◯	<60	Predator/Scavenger	S/H/B	Crawler/Walker	3 to 5	90	−1 to 35	1.4 to 54	Sexual (int)	Planktotrophic	Hull/Ballast	https://www.marlin.ac.uk/	
*Mytilus galloprovincialis*	Mediterranean mussel	◯	◯	<20	Filter/suspension	S/H/B	Sessile/Crawler	<2	40	3 to 25	10 to 38	Sexual (ext)	Planktotrophic	Hull	Degen and Faulwetter (2019)	
*Ascidiella aspersa*	European sea squirt	◯	◯	<20	Filter/suspension	H/B	Sessile	<2	<2	3 to 26	12 to 40	Asexual/Sexual (ext)	Lecithotrophic	Hull	Degen and Faulwetter (2019)	https://invasions.si.edu/nemesis
*Amphibalanus amphitrite*	Striped barnacle		◯	<20	Filter/suspension	H/B	Sessile	1 to 5	17	1.5 to 40	10 to 52	Sex brooding	Planktotrophic	Hull	https://invasions.si.edu/nemesis	
*Balanus glandula*	Barnacle		◯	<20	Filter/suspension	H	Sessile	7 to 10	28	−2 to 35	14 to 70+	Sexual (int)/broadcast spawner	Planktotrophic	Hull	Degen and Faulwetter (2019)	https://invasions.si.edu/nemesis
*Codium fragile subsp. Fragile*	Green sea fingers—algae	◯	◯	<20	Photosynthetic	H/B	Sessile	1	—	−2 to 30	12 to 42	Sexual (ext)/sporogenesis/vegetative	Gametophytic plankton	Hull	https://invasions.si.edu/nemesis	
*Ciona intestinalis*	Yellow sea squirt	◯		<1000	Filter/suspension	H/B	Sessile	2 to 5	7	0 to 27	12 to 40	Sexual (ext)	Lecithotrophic	Hull	Degen and Faulwetter (2019)	https://invasions.si.edu/nemesis
*Halicarcinus planatus*	Decapod	◯		<270	Deposit feeder	S/H/B	Crawler/Walker	<2	80	2 to 17	5 to 60	Sex brooding	Planktotrophic	Other/Ballast/Hull	https://www.sealifebase.se/	https://invasions.si.edu/nemesis
*Bugula neritina*	Ruby bryozoan	◯		<320	Filter/suspension	H/B	Sessile	<2	<1	4 to 30	18 to 40	Asexual/Sex brooding	Lecithotrophic	Hull	Degen and Faulwetter (2019)	https://invasions.si.edu/nemesis
*Austromininus modestus*	Darwin's barnacle	◯		<20	Filter/suspension	H/B	Sessile	<2	40	4 to 21	14 to 47	Sexual (int)	Planktotrophic	Hull/Other	https://www.marlin.ac.uk/	

*Note*: Temperatures and salinity levels based on recorded or projected species survival, rather than upper and lower reproductive/developmental thresholds (as data is limited).

^a^ Details including most likely potential pathways of arrival and the list is ranked by potential to arrive, establish, and pose a threat through biodiversity and/or economic impacts, based on (Roy et al., 2019). Primary routes of transport also shown.

^b^ *Mytilus chilensis* based on close relative *Mytilus edulis* due to limited species‐specific knowledge. Traits amalgamated from Degen and Faulwetter (2019), https://invasions.si.edu/nemesis, https://www.marlin.ac.uk/, and https://www.sealifebase.se/.

## 4 DISCUSSION

Hull biofouling is a major issue for both the maritime industry and environmental managers, as it increases ship drag and corrosion while also acting as a direct vector for dispersal of non‐native marine species (Davidson et al., 2016). Dispersal of non‐native species via this vector is relatively high as organisms can last long periods attached to hulls and often have time to develop in warmer waters before the journey South (Hughes & Ashton, 2017; Lewis et al., 2003). While modern anti‐fouling coatings can reduce the likelihood of attachment considerably, a number of species are still potentially able to persist, particularly in protected niche areas such as shafts and sea chests, on any uncoated surfaces, and on vessels that are not regularly maintained (Davidson et al., 2016; Frey et al., 2014; Lee & Chown, 2007). A range of other factors can influence survivorship of attached communities, including vessel movement behaviour and environmental conditions, as well as hull surface scour from turbulence of fast‐moving vessels (Coutts et al., 2010; Lewis et al., 2003) and scour from ice (Hughes & Ashton, 2017; Lee & Chown, 2009; Lewis et al., 2003). Historically these factors alongside lower sea temperature and extensive ice cover have likely shielded SGSSI. However, there have been recordings of species surviving despite transit scour. Primarily this is where they are positioned on hard‐to‐reach protected *niche* areas, or where ice cover and/or thickness has receded, as it has around the Antarctic and sub‐Antarctic region (Chown et al., 2012; Coutts et al., 2010; Hughes & Ashton, 2017; Stammerjohn et al., 2012).

Our analysis highlighted passenger vessels, fishing vessels, offshore survey vessels and pleasure yachts as highest priority vectors of threat for the potential introduction of marine non‐native species, relative to other vessel types in this analysis (Table 2). These vessels are all relatively numerous, of a mid to large size (indicating high WSA), are active throughout most of the year, and stop at multiple ports whilst frequently crossing international waters. Vessels also typically originate from a range of international ports, predominantly within the Scotia Sea and Magellanic ecoregions (namely within areas holding species acclimatised to similar environmental conditions).

Length of time sitting stationary in port before extended voyages is a key factor governing biofouling accumulation and establishment. The scour, cavitation, and turbulence from frequent fast movement will reduce the likelihood of both initial hull settlement and survival once settled (Coutts et al., 2010). Vessels which have prolonged stationary periods followed by multiple occasional journeys to a number of locations, such as yachts and fishing vessels, are therefore more likely to introduce biofouling species (McCarthy et al., 2019; Williams et al., 2013).

In this context, the likelihood of introductions from hull biofouling is broadly equal across commercial and recreational vessels (Williams et al., 2013). However, some individual vessels will have greater funds available to conduct maintenance on a periodic schedule or will be incentivised through improved aesthetic appearance for customers and efficiency of travel (Davidson et al., 2016). The trade‐off between the streamlining benefits to the hull through regular maintenance and the cost of such maintenance to owners, will likely be the primary decision governing current levels of biofouling on each vessel in the absence of specific regulation. Furthermore, niche areas (i.e. inaccessible parts of a vessel's underwater surface more susceptible to biofouling, such as sea chests, propellers, etc.) on all vessels tend to accumulate and protect species and are often missed in basic cleaning (Davidson et al., 2016). Internal seawater systems (here considered distinct from, though connected to, sea chests) can also house high densities of marine non‐native species but can be difficult and expensive to monitor and clean and are therefore often neglected (Davidson et al., 2021). This makes them another under‐researched potential route for spread of species.

Most of the vessels in this analysis came to South Georgia via intermediate stops in nearby regional locations such as South America. Introductions from other identified distant areas such as Europe or Asia, had lower vessel numbers, extended travel time and varying environmental conditions to traverse, so are considered lower threat. However, these vessels cannot be discounted entirely as some indirect routes may play an important role in spreading marine non‐native species (Saebi et al., 2020). These distant vessels visited SGSSI frequently or annually and typically stayed anchored for long periods at ports and inshore areas, so may still be important vectors.

Within South Georgia itself, the location with the highest likelihood of introductions was KEP. KEP is the territory's administrative centre, and port at which all vessels are required to call to complete Customs clearance for SGSSI waters. Stationary vessels were clustered in seven distinct locations around the wider Cumberland Bay area adjoining KEP, primarily at Grytviken, east of KEP and north of the Greene Peninsula. KEP was also identified as an important dispersion hub to other ports for any potential non‐native species as all vessels visiting South Georgia, or fishing within its waters *must* call at KEP at some point in their visit. Mandatory customs visit compliance is currently considered to be 100%. However, vessels transiting through SGSSI waters do not need to report to KEP, but must stay outside the territory's 12 nm limit, and therefore present a lower threat.

The busy anchorages of the Bay of Isles, Gold Harbour, St Andrews Bay and Stromness Bay, which have a number of route connections to other ports, will be important locations to monitor over time to assess whether introductions have occurred, and to alert management authorities to stop further spread. Importantly, the *initial* inshore anchorage within South Georgia made by new vessels is not always at the mandatory stop of KEP, particularly for Pleasure yachts and passenger cruise ships. Other locations around the island are also being frequently used as initial entry stops prior to arrival at KEP. Initial introduction to any of these identified priority locations is likely to lead to rapid spread to other locations due to the frequent transit of vessels from here to multiple other SGSSI locations.

### 4.1 Existing regional knowledge and legislation

The initial non‐native species management prioritisation work completed by Roy et al. (2019) adapted in Table 3, highlighted the likeliest novel (terrestrial and marine) species arrivals into SGSSI using the existing limited knowledge for the territory. These species are considered (through expert opinion) to be most likely to arrive, establish and impact the territory. Following the work, the Government of SGSSI implemented a new ‘Biosecurity Audit’ system over the 2018/2019 season to check the biosecurity procedures of visiting vessels, aiming to help facilitate effective biosecurity checks before arrival to SGSSI. Subsequently the Non‐native Species Secretariat also identified remaining gaps here and in the wider UKOTs (Key & Moore, 2019), and made recommendations for strengthening the biosecurity systems of each territory (Government of South Georgia & the South Sandwich Islands, 2019; Key, 2018). However, while terrestrial invasive species mitigation and ballast exchange protocols are in place, there is no similar current mitigation for reducing the risk of marine introductions from the hulls or internal seawater systems of visiting vessels. This should therefore be a management priority.

The likelihood of introduction and dispersal potential of the non‐native species associated with vessel biofouling are governed by a range of key factors such as condition, frequency of maintenance of the vessel and direction of travel (Lewis et al., 2003; Sylvester et al., 2011). In response to these known risk factors, the IMO created broad internationally relevant guidelines for the control and management of ship biofouling to minimise invasive species introductions and spread (International Maritime Organisation, 2023). These guidelines are further supported at the regional level by the IMO Polar code (International Maritime Organisation, 2017). However, this guidance does not currently require mandatory cleaning before entry to the Antarctic region (including SGSSI). The guidelines currently only recommend creation of a biofouling management plan, keeping a record book, and installation and maintenance of anti‐fouling systems. Regular in‐water inspection, cleaning, and maintenance of ship hulls and submerged surfaces/systems is also advocated, but is not required or time‐bound (International Maritime Organisation, 2023). Separate guidelines exist for smaller recreational vessels <24 m in length (International Maritime Organisation, 2012). This leaves broad scope for improvement of these regulations.

### 4.2 Management recommendations and future research

This analysis uses AIS data of movement, behaviour and hull specifications of vessels entering SGSSI waters. Threat assessments are therefore based on a scenario where hull condition and maintenance are assumed poor enough for all vessels to facilitate the introduction of non‐native species. Some individuals or general vessel types will be in better or worse condition than others. True condition will therefore strongly weigh an increased threat towards those vessels which have poor maintenance, even if their behaviour and specification is considered relatively less likely to introduce non‐native species. Assessment of typical real‐world levels of compliance, maintenance and hull condition for each vessel type is therefore essential.

Relatively little is known about the full diversity of existing native species found around the SGSSI archipelago and their natural extent (Barnes et al., 2006; Brewin & Brickle, 2010; Convey & Peck, 2019; Glon et al., 2020; Hogg et al., 2011). Baseline data are essential to highlight new non‐native species and predict and manage their effect on native systems. As a key next step, we recommend better characterisation of the native baseline fauna and flora, allowing detection and monitoring of emergent non‐native species. Williams et al. (2013) shows that reducing the risk from biofouling and ballast release requires managing both large and small crafts, from both commercial and recreational settings. These vessels appear to broadly carry the same typical biofouling accumulation loads and percent of non‐native species. The danger of assuming that smaller vessels are negligible risk, or that commercially managed boats are better maintained, allows these unquantified pathways to remain hazardous (Williams et al., 2013; Zabin et al., 2014). Instead, most known established non‐native species are associated with multiple vectors (Williams et al., 2013). Therefore, future introductions could be reduced by assessing all the vectors by creating a prioritisation framework based on these multiple factors (Castro et al., 2021; Davidson et al., 2017; Williams et al., 2013).

Due to the limited resources available and increasing activity across the region, essential management should prioritise effective management and conservation actions (Giakoumi et al., 2019; Hiscock et al., 2013), and routes and sites most likely to be facilitating or receiving non‐native species introductions (McGeoch et al., 2016). This process will likely require decision‐analysis to play‐off multiple options, including costs and practicality, until more data are known for vessel conditions and species presence (Adem Esmail & Geneletti, 2018; Booy et al., 2017). For instance, vessels travelling from local to intermediate distances tend to have the highest likelihood of introduction success (Seebens et al., 2013), and vessels from similar environments are more likely to carry organisms that survive transit and establish once arrived (Holland et al., 2021; Keller et al., 2011). Within this analysis' context, ‘Local’ areas (i.e. ~2–4000 km distance), would include Argentina, Chile, Uruguay, Brazil, South Africa. ‘Intermediate’ areas (i.e. ~8–10,000 km distance), would be temperate African, Mediterranean and Caribbean regions. The identified high‐threat vessel types in our analysis, such as passenger vessels, travelling from these local similar environment locations such as Patagonian South America and the Falkland Islands, will be a management and monitoring priority. International and cross‐territory collaboration will therefore be a key component of making such management decisions effective.

Little is currently known on potential non‐native species‐specific physiological tolerances to environmental changes (or ability to reproduce), either during transit to, or within the environmental extremes found within SGSSI and the sub‐Antarctic (e.g. Convey & Peck, 2019; Davenport & Macalister, 1996; Holland et al., 2021; Navarro et al., 2024; Peck et al., 2004, 2014). These data will be a key next step in order to identify riskiest ports of origin, based on species known or predicted to come from certain locations. Physiological tolerance data would also help ascertain which species are able to survive both the journey and new environment, and the likely consequences of their introduction to SGSSI biodiversity.

Holland et al. (2021) suggest that likelihood of hull fouling species surviving in the environmentally similar shallow benthic habitats near Australia's East Antarctica locations, are currently very low, but plausible. Four species (*Asterias amurensis*, *Geukensia demissa*, *Hypnea musciformis* and *Undaria pinnatifida*) of the 33 analysed were identified as potential current threats, and five species (adding *Charybdis japonica*) were identified as threats under future modelled climate change (Holland et al., 2021). Holland et al. (2021) further noted that other invasive species, such as *Carcinus maenas* (also identified as likely threats to SGSSI), have the ability to adapt to cold conditions well below those experienced in its native range, and therefore future modelling predictions are likely to underestimate threat from highly plastic species. More broadly, improved knowledge of species' life‐history characteristics (e.g. reproductive thermal tolerance, life span, dispersal potential), is critical to our ability to better manage, predict and mitigate their threat (Costello et al., 2015; López‐Farrán et al., 2021).

Looking to the future within a rapidly changing environment, we also need to be able to project future environmental conditions in the territories over the short‐ to mid‐term, to assess which new species are likely to *become* threats from vessel introductions. This future research would need to include the fundamental niches and potential distribution of both: (1) known regional species extending their range to SGSSI and (2) non‐native species (i.e. within the Scotia Arc, Antarctica, South America and South Africa), in order to determine likelihood of natural introductions as conditions change.

#### 4.2.1 Biosecurity monitoring

Beyond predictions, the ability to rapidly detect and identify any new arrivals is essential for appropriate threat mitigation. Monitoring at the identified connected ports with highest level of threat would likely come in the form of focussed vessel inspection based on identified threat characteristics, assessment of biofouling and ballast water management documentation, and diver‐based/Remotely Operated Vehicle surveys of hulls and niche areas (Zabin et al., 2018). Such monitoring and pre‐emptive actions have been used to good effect in countries such as New Zealand and USA (Hawaii) where risks from marine introductions are broadly similar (Georgiades et al., 2020; Zabin et al., 2018). However, it should be noted that in SGSSI itself, this kind of monitoring outside of KEP would be complex and logistically difficult to achieve.

### 4.3 Study limitations

The factors used in this analysis were chosen based on existing knowledge of vessel activity behaviour and ship design that is considered to increase the likelihood of introducing non‐native marine species to an environment. However, a range of additional factors exist, such as the environmental conditions at the origin and destination ports and individual species' physiological tolerances. These additional factors were outside this analysis but will of course affect the overall likelihood of non‐native species' initial arrival and establishment now and as environmental conditions change (Davidson et al., 2017; Hughes et al., 2020).

Smaller‐sized yachts (pleasure vessels) active within our study region are not required to transmit AIS, and are therefore missed from the overall assessment. The threat associated with yachts (pleasure vessels) will therefore likely be higher. Similarly, AIS signals can be deliberately switched on and off, or put into ‘receive mode’, for example, by patrol vessels, tenders, port pilot vessels, or illegal operators, or can be unintentionally lost through adverse conditions interfering with the GPS. These intermittent or lost signals, while rare, can cause analysis gaps or confusing analysis outputs. Some smaller yachts do not use AIS at all, causing gaps in our knowledge of their full movement and behaviour, which can only be supplemented by (more simplified) port records. Vessel AIS attributes also have the potential to change through time (i.e. vessel name and vessel type designations), or be incorrectly entered into the AIS database, meaning that these data must be treated with caution, and a degree of scepticism.

Finally, the vessels in this analysis are assumed to comply with international ballast water exchange regulations, however there is scope for emergency release, or non‐compliance from some vessels.

#### 4.3.1 Summary and recommendations

Initial management actions to mitigate the threat of non‐native species could include introducing marine biosecurity measures as conditions of entry on fishing licences and visit permits for vessels entering SGSSI. Options applied would depend on feasibility, however measures might include a pre‐arrival inspection at a gateway port, or a requirement for the first port‐of‐call on entering the SGSSI Maritime Zone to be KEP, if vessels are stopping inshore. This would allow vessel hull, internal seawater systems and ballast‐system state to be assessed. This would further limit any potential spread to one location (KEP) and would allow quarantine if needed (Hewitt & Campbell, 2007). Additional standards could be introduced to lower the likelihood of established biofouling communities arriving (Davidson et al., 2016). This might require hull cleaning to have been conducted within a set time‐period, or random inspections on high‐threat vessels before entry to inshore waters. This will be for the government of SGSSI to decide on details, however they may wish to follow similar voluntary or mandatory best‐practice guidelines from other nearby or similar countries such as Chile, New Zealand and Australia (see GEF‐UNDP‐IMO, 2022 for a summary of guidelines). Australia for instance has relatively strict rules requiring vessels to have been cleaned of all biofouling within 30 days of arriving (DAWE, 2022). This also includes an active biofouling management plan and record book, and regular antifouling renewal schedule. Hull cleaning could more broadly be specified to a set international standard, protocol or certification (when developed) and evidenced in logbooks as per current IMO ballast rules.

Mandatory customs check questions could be relatively easily expanded to include the records of each boat's history regarding last cleaning, antifouling application, recent activities and detailed trip locations within SGSSI (rather than just the current previous and next port of call requirement). This would further allow pre‐border risk assessment to be conducted. Similarly, while the likelihood of introducing non‐native species is relatively low for tenders, new rules may request tender hulls to have been cleaned when stowed before initially entering SGSSI inshore waters.

Optimally, these requirements would eventually meet an internationally accepted biosecurity compliance standard, regardless of flag state. These standards may potentially follow existing practice in similar archipelagos such as New Zealand, Hawaii or the Galapagos, adapting where necessary to mitigate local threats (see Georgiades et al., 2020 & GEF‐UNDP‐IMO, 2022 for further details on national, international and regional biofouling regulation and management practices). Standards would need to be comprehensive to cover the risks associated with South Georgia and beyond (i.e. a regional collaborative management approach), and be feasible for enforcement before entering inshore waters. One potential option for government enforcement would then be to state mandatory compliance and enhanced procedures such as regular hull cleaning within regional agreements such as the IMO/Polar code biofouling guidelines (IMO, 2017), before entry to SGSSI waters. This would allow pre‐emptive risk reduction and improve data for future management or additional tougher interventions.

In the longer‐term, monitoring and assessment of vessels and benthos in key locations would begin to allow detection of any existing occurrence of identified high‐risk species and would establish benthic baselines. A priority for this work would be long‐term monitoring at KEP. Regular site‐prioritised monitoring of identified anchorages in the ports with the next highest likelihood of receiving non‐native species would also be beneficial, but incur high cost. Periodic assessment of the state of the hull from randomly chosen vessels would further help prioritise high‐threat vessels. In addition to hull checks, the current *Port visit reports* should require greater detail on *all* recent stops taken, rather than only the immediate previous and final destinations currently required.

All such management will require cross‐territory and regional collaboration to ensure that particularly high‐threat vessels are frequently monitored and assessed for biofouling extent before they enter into the region (McCarthy et al., 2022; McDonald et al., 2020). If vessels were required to submit biofouling management plans to authorities in SGSSI as well as key regional ports, for example, Ushuaia and Port Stanley, high‐threat vessels could be identified well in advance of their arrival in SGSSI. Further, stronger biosecurity across nations in South America and the South Atlantic would encourage greater adoption of and compliance regarding biosecurity. In all the cases, pre‐emptive measures, which are prioritised based on risk and initiated *before* arrival, are the key to limiting the likelihood of spread and establishment of non‐native species in this highly sensitive environment (Booy et al., 2017; Dawson et al., 2022; Hogg et al., 2011). These islands' high biodiversity, endemicity and position as a key transport gateway into the Antarctic wilderness region make it a management priority (McCarthy et al., 2019).

## AUTHOR CONTRIBUTIONS

**Daniel T. I. Bayley:** Conceptualization (equal); data curation (lead); formal analysis (lead); investigation (lead); methodology (lead); project administration (lead); resources (lead); software (lead); validation (lead); visualization (lead); writing – original draft (lead); writing – review and editing (equal). **Paul E. Brewin:** Conceptualization (equal); formal analysis (supporting); funding acquisition (supporting); supervision (supporting); validation (supporting); visualization (supporting); writing – original draft (supporting); writing – review and editing (equal). **Ross James:** Conceptualization (equal); writing – review and editing (equal). **Arlie H. McCarthy:** Investigation (supporting); validation (supporting); writing – review and editing (equal). **Paul Brickle:** Conceptualization (equal); data curation (supporting); funding acquisition (lead); methodology (supporting); project administration (supporting); resources (lead); supervision (supporting); validation (supporting); visualization (supporting); writing – original draft (supporting); writing – review and editing (equal).

## CONFLICT OF INTEREST STATEMENT

There are no known conflicts of interest.

## STATEMENT ON INCLUSION

Our study brings together authors from a number of different countries, including scientists based in the territories where the study was carried out.

## Supporting information


Appendix S1.

## Data Availability

The data used within this analysis contains confidential information about private vessels and their movements (through AIS transmission) and so cannot be made freely available for public view. GIS data and code available on request via the Falkland Islands Data Portal: http://dataportal.saeri.org/.

## References

[ece311299-bib-0001] Adem Esmail, B. , & Geneletti, D. (2018). Multi‐criteria decision analysis for nature conservation: A review of 20 years of applications. Methods in Ecology and Evolution, 9(1), 42–53.

[ece311299-bib-0002] Avila, C. , Angulo‐Preckler, C. , Martín‐Martín, R. P. , Figuerola, B. , Griffiths, H. J. , & Waller, C. L. (2020). Invasive marine species discovered on non–native kelp rafts in the warmest Antarctic Island. Scientific Reports, 10, 1639. 10.1038/s41598-020-58561-y 32005904 PMC6994651

[ece311299-bib-0003] Bailey, S. A. , Brown, L. , Campbell, M. L. , Canning‐Clode, J. , Carlton, J. T. , Castro, N. , Chainho, P. , Chan, F. T. , Creed, J. C. , Curd, A. , Darling, J. , Fofonoff, P. , Galil, B. S. , Hewitt, C. L. , Inglis, G. J. , Keith, I. , Mandrak, N. E. , Marchini, A. , Mckenzie, C. H. , … Zhan, A. (2020). Trends in the detection of aquatic non‐indigenous species across global marine, estuarine and freshwater ecosystems: A 50‐year perspective. Diversity and Distributions, 26, 1780–1797. 10.1111/ddi.13167 36960319 PMC10031752

[ece311299-bib-0004] Bakker, J. , & van Vlaardingen, P. L. A. (2017). Wetted surface area of recreational boats: RIVM report 2017‐0116. Available from: https://www.rivm.nl/bibliotheek/rapporten/2017‐0116.pdf

[ece311299-bib-0005] Barnes, D. K. A. , Linse, K. , Waller, C. , Morely, S. , Enderlein, P. , Fraser, K. P. P. , & Brown, M. (2006). Shallow benthic fauna communities of South Georgia Island. Polar Biology, 29, 223–228. 10.1007/s00300-005-0042-0

[ece311299-bib-0006] Bax, N. , Carlton, J. T. , Mathews‐Amos, A. , Haedrich, R. L. , Howarth, F. G. , Purcell, J. E. , Rieser, A. , & Gray, A. (2001). The control of biological invasions in the world’s oceans. Conservation Biology, 15, 1234–1246. 10.1111/j.1523-1739.2001.99487.x

[ece311299-bib-0007] Bax, N. , Williamson, A. , Aguero, M. , Gonzalez, E. , & Geeves, W. (2003). Marine invasive alien species: A threat to global biodiversity. Marine Policy, 27, 313–323. 10.1016/S0308-597X(03)00041-1

[ece311299-bib-0008] Blackburn, T. M. , Pyšek, P. , Bacher, S. , Carlton, J. T. , Duncan, R. P. , Jarošík, V. , Wilson, J. R. U. , & Richardson, D. M. (2011). A proposed unified framework for biological invasions. Trends in Ecology & Evolution, 26, 333–339. 10.1016/j.tree.2011.03.023 21601306

[ece311299-bib-0009] Booy, O. , Mill, A. C. , Roy, H. E. , Hiley, A. , Moore, N. , Robertson, P. , Baker, S. , Brazier, M. , Bue, M. , Bullock, R. , Campbell, S. , Eyre, D. , Foster, J. , Hatton‐Ellis, M. , Long, J. , Macadam, C. , Morrison‐Bell, C. , Mumford, J. , Newman, J. , … Wyn, G. (2017). Risk management to prioritise the eradication of new and emerging invasive non‐native species. Biological Invasions, 19, 2401–2417. 10.1007/s10530-017-1451-z

[ece311299-bib-0010] Brasier, M. J. , Barnes, D. , Bax, N. , Brandt, A. , Christianson, A. B. , Constable, A. J. , Downey, R. , Figuerola, B. , Griffiths, H. , Gutt, J. , Lockhart, S. , Morley, S. A. , Post, A. L. , Van de Putte, A. , Saeedi, H. , Stark, J. S. , Sumner, M. , & Waller, C. L. (2021). Responses of Southern Ocean seafloor habitats and communities to global and local drivers of change. Frontiers in Marine Science, 8, 109. 10.3389/fmars.2021.622721

[ece311299-bib-0011] Brewin, P. , & Brickle, P. (2010). Invasive species monitoring of South Georgia. Shallow Marine Survey Group. Report for JNCC, 1–12. Avaiable from: https://smsg‐falklands.org/images/Reports_Publications/sg%20invasive%20species%20report%202010.pdf

[ece311299-bib-0012] Cárdenas, L. , Leclerc, J. C. , Bruning, P. , Garrido, I. , Détrée, C. , Figueroa, A. , Astorga, M. , Navarro, J. M. , Johnson, L. E. , Carlton, J. T. , & Pardo, L. (2020). First mussel settlement observed in Antarctica reveals the potential for future invasions. Scientific Reports, 10, 1–8. 10.1038/s41598-020-62340-0 32218472 PMC7099062

[ece311299-bib-0013] Castro, K. L. , Battini, N. , Giachetti, C. B. , Trovant, B. , Abelando, M. , Basso, N. G. , & Schwindt, E. (2021). Early detection of marine invasive species following the deployment of an artificial reef: Integrating tools to assist the decision‐making process. Journal of Environmental Management, 297, 113333. 10.1016/j.jenvman.2021.113333 34329910

[ece311299-bib-0014] Chown, S. L. , Huiskes, A. H. L. , Gremmen, N. J. M. , Lee, J. E. , Terauds, A. , Crosbie, K. , Frenot, Y. , Hughes, K. A. , Imura, S. , Kiefer, K. , Lebouvier, M. , Raymond, B. , Tsujimoto, M. , Ware, C. , Van De Vijver, B. , & Bergstrom, D. M. (2012). Continent‐wide risk assessment for the establishment of nonindigenous species in Antarctica. Proceedings of the National Academy of Sciences of the United States of America, 109, 4938–4943. 10.1073/pnas.1119787109 22393003 PMC3323995

[ece311299-bib-0015] Clarke, A. , Barnes, D. K. A. , & Hodgson, D. A. (2005). How isolated is Antarctica? Trends in Ecology & Evolution, 20, 1–3. 10.1016/j.tree.2004.10.004 16701330

[ece311299-bib-0016] Convey, P. , & Peck, L. S. (2019). Antarctic environmental change and biological responses. Science Advances, 5, eaaz0888. 10.1126/sciadv.aaz0888 31807713 PMC6881164

[ece311299-bib-0017] Costello, M. J. , Claus, S. , Dekeyzer, S. , Vandepitte, L. , Tuama, É. , Lear, D. , & Tyler‐Walters, H. (2015). Biological and ecological traits of marine species. PeerJ, 2015, 1–29. 10.7717/peerj.1201 PMC4548538 26312188

[ece311299-bib-0018] Coutts, A. D. M. , Piola, R. F. , Taylor, M. D. , Hewitt, C. L. , & Gardner, J. P. A. (2010). The effect of vessel speed on the survivorship of biofouling organisms at different hull locations. Biofouling, 26, 539–553. 10.1080/08927014.2010.492469 20526914

[ece311299-bib-0019] Csardi, G. , & Nepusz, T. (2006). The igraph software package for complex network research. InterJournal, Complex Systems, 1695, 1–9.

[ece311299-bib-0020] Davenport, J. , & Macalister, H. (1996). Environmental conditions and physiological tolerances of intertidal fauna in relation to shore zonation at Husvik, South Georgia. Journal of the Marine Biological Association of the United Kingdom, 76, 985–1002. 10.1017/S0025315400040923

[ece311299-bib-0021] Davidson, A. , Fusaro, A. , Sturtevant, R. , & Kashian, D. (2017). Development of a risk assessment framework to predict invasive species establishment for multiple taxonomic groups and vectors of introduction. Management of Biological Invasions, 8, 25–36. 10.3391/mbi.2017.8.1.03

[ece311299-bib-0022] Davidson, I. , Cahill, P. , Hinz, A. , Kluza, D. , Scianni, C. , & Georgiades, E. (2021). A review of biofouling of ships' internal seawater systems. Frontiers in Marine Science, 8, 1–16. 10.3389/fmars.2021.761531 37369526

[ece311299-bib-0023] Davidson, I. C. , Scianni, C. , Hewitt, C. , Everett, R. , Holm, E. , Tamburri, M. , & Ruiz, G. (2016). Mini‐review: Assessing the drivers of ship biofouling management—Aligning industry and biosecurity goals. Biofouling, 32, 411–428. 10.1080/08927014.2016.1149572 26930397

[ece311299-bib-0024] DAWE . (2022). Australian biofouling management requirements (version 1), Department of Agriculture, Water and the Environment, Canberra, Australia. Available from: https://www.agriculture.gov.au/sites/default/files/documents/Australian‐biofouling‐management‐requirements.pdf

[ece311299-bib-0025] Dawson, W. , Peyton, J. M. , Pescott, O. L. , Adriaens, T. , Cottier‐Cook, E. J. , Frohlich, D. S. , Key, G. , Malumphy, C. , Martinou, A. F. , Minchin, D. , Moore, N. , Rabitsch, W. , Rorke, S. L. , Tricarico, E. , Turvey, K. M. A. , Winfield, I. J. , Barnes, D. K. A. , Baum, D. , Bensusan, K. , … Roy, H. E. (2022). Horizon scanning for potential invasive non‐native species across the United Kingdom overseas territories. Conservation Letters, 16, e12928. 10.1111/conl.12928

[ece311299-bib-0026] Degen, R. , & Faulwetter, S. (2019). The Arctic traits database—A repository of Arctic benthic invertebrate traits. Earth System Science Data, 11, 301–322. 10.5194/essd-11-301-2019

[ece311299-bib-0027] Dulière, V. , Guillaumot, C. , Lacroix, G. , Saucède, T. , López‐Farran, Z. , Danis, B. , Schön, I. , & Baetens, K. (2022). Dispersal models alert on the risk of non‐native species introduction by ballast water in protected areas from the Western Antarctic peninsula. Diversity and Distributions, 28, 649–666. 10.1111/ddi.13464

[ece311299-bib-0028] Fraser, C. I. , Morrison, A. K. , Hogg, A. M. C. , Macaya, E. C. , van Sebille, E. , Ryan, P. G. , Padovan, A. , Jack, C. , Valdivia, N. , & Waters, J. M. (2018). Antarctica's ecological isolation will be broken by storm‐driven dispersal and warming. Nature Climate Change, 8, 704–708. 10.1038/s41558-018-0209-7

[ece311299-bib-0029] Frenot, Y. , Chown, S. L. , Whinam, J. , Selkirk, P. M. , Convey, P. , Skotnicki, M. , & Bergstrom, D. M. (2005). Biological invasions in the Antarctic: Extent, impacts and implications. Biological Reviews, 80, 45–72. 10.1017/S1464793104006542 15727038

[ece311299-bib-0030] Frey, M. A. , Simard, N. , Robichaud, D. D. , Martin, J. L. , & Therriault, T. W. (2014). Fouling around: Vessel sea‐chests as a vector for the introduction and spread of aquatic invasive species. Management of Biological Invasions, 5, 21–30. 10.3391/mbi.2014.5.1.02

[ece311299-bib-0031] GEF‐UNDP‐IMO . (2022). GloFouling partnerships project and GIA for marine biosafety: Compilation and comparative analysis of existing and emerging regulations, Standards and Practices Related to Ships' Biofouling Management. Report available from: https://www.glofouling.imo.org/publications‐menu

[ece311299-bib-0032] Georgiades, E. , Kluza, D. , Bates, T. , Lubarsky, K. , Brunton, J. , Growcott, A. , Smith, T. , McDonald, S. , Gould, B. , Parker, N. , & Bell, A. (2020). Regulating vessel biofouling to support New Zealand's marine biosecurity system—A blue print for evidence‐based decision making. Frontiers in Marine Science, 7, 1–15. 10.3389/fmars.2020.00390 32802822

[ece311299-bib-0033] Giakoumi, S. , Katsanevakis, S. , Albano, P. G. , Azzurro, E. , Cardoso, A. C. , Cebrian, E. , Deidun, A. , Edelist, D. , Francour, P. , Jimenez, C. , Mačić, V. , Occhipinti‐Ambrogi, A. , Rilov, G. , & Sghaier, Y. R. (2019). Management priorities for marine invasive species. Science of the Total Environment, 688, 976–982. 10.1016/j.scitotenv.2019.06.282 31726580

[ece311299-bib-0034] Glon, H. , Costa, M. , de Lecea, A. M. , Goodwin, C. , Cartwright, S. , Díaz, A. , Brickle, P. , & Brewin, P. E. (2020). First record of the plumose sea anemone, metridium senile (Linnaeus, 1761), from The Falkland Islands. BioInvasions Records, 9, 461–470. 10.3391/bir.2020.9.3.02

[ece311299-bib-0035] Government of South Georgia & the South Sandwich Islands . (2019). Biosecurity Handbook 2020–2021. Available from: https://www.gov.gs/docsarchive/Environment/Biosecurity/Biosecurity_Handbook.pdf

[ece311299-bib-0036] Griffiths, H. J. , Barnes, D. K. A. , & Linse, K. (2009). Towards a generalized biogeography of the Southern Ocean benthos. Journal of Biogeography, 36, 162–177. 10.1111/j.1365-2699.2008.01979.x

[ece311299-bib-0037] Griffiths, H. J. , & Waller, C. L. (2016). The first comprehensive description of the biodiversity and biogeography of Antarctic and sub‐Antarctic intertidal communities. Journal of Biogeography, 43, 1143–1155. 10.1111/jbi.12708

[ece311299-bib-0038] Hewitt, C. L. , & Campbell, M. L. (2007). Mechanisms for the prevention of marine bioinvasions for better biosecurity. Marine Pollution Bulletin, 55, 395–401. 10.1016/j.marpolbul.2007.01.005 17379259

[ece311299-bib-0039] Hiscock, K. , Bayley, D. T. I. , Pade, N. , Lacey, C. , Cox, E. , & Enever, R. (2013). Prioritizing action for recovery and conservation of marine species: A case study based on species of conservation importance around England. Aquatic Conservation: Marine and Freshwater Ecosystems, 23, 88–110. 10.1002/aqc.2283

[ece311299-bib-0040] Hogg, O. T. , Barnes, D. K. A. , & Griffiths, H. J. (2011). Highly diverse, poorly studied and uniquely threatened by climate change: An assessment of marine biodiversity on South Georgia's continental shelf. PLoS One, 6, e19795. 10.1371/journal.pone.0019795 21647236 PMC3102052

[ece311299-bib-0041] Holland, O. , Shaw, J. , Stark, J. S. , & Wilson, K. A. (2021). Hull fouling marine invasive species pose a very low, but plausible, risk of introduction to East Antarctica in climate change scenarios. Diversity and Distributions, 27, 973–988. 10.1111/ddi.13246

[ece311299-bib-0043] Hughes, K. A. , & Ashton, G. V. (2017). Breaking the ice: The introduction of biofouling organisms to Antarctica on vessel hulls. Aquatic Conservation: Marine and Freshwater Ecosystems, 27, 158–164. 10.1002/aqc.2625

[ece311299-bib-0044] Hughes, K. A. , Pescott, O. L. , Peyton, J. , Adriaens, T. , Cottier‐Cook, E. J. , Key, G. , Rabitsch, W. , Tricarico, E. , Barnes, D. K. A. , Baxter, N. , Belchier, M. , Blake, D. , Convey, P. , Dawson, W. , Frohlich, D. , Gardiner, L. M. , González‐Moreno, P. , James, R. , Malumphy, C. , … Roy, H. E. (2020). Invasive non‐native species likely to threaten biodiversity and ecosystems in the Antarctic peninsula region. Global Change Biology, 26, 2702–2716. 10.1111/gcb.14938 31930639 PMC7154743

[ece311299-bib-0045] International Maritime Organisation . (2004). International convention for the control and management of ships' ballast water and sediments. BWM/CONF/36, 36. Available from http://library.arcticportal.org/1913/1/International%20Convention%20for%20the%20Control%20and%20Management%20of%20Ships%27%20Ballast%20Water%20and%20Sediments.pdf

[ece311299-bib-0046] International Maritime Organisation . (2007). Guidelines for ballast water exchange in the Antarctic Treaty Area. Annex 4. Available from: https://wwwcdn.imo.org/localresources/en/KnowledgeCentre/IndexofIMOResolutions/MEPCDocuments/MEPC.163(56).pdf

[ece311299-bib-0047] International Maritime Organisation . (2012). Guidance for minimizing the transfer of invasive aquatic species as biofouling (hull fouling) for recreational craft. MEPC.1/Circ. 792. Available from: https://wwwcdn.imo.org/localresources/en/OurWork/Environment/Documents/MEPC.1‐Circ.792.pdf

[ece311299-bib-0048] International Maritime Organisation . (2015) Revised guidelines for the onboard operational use of shipborne automatic identification systems (AIS). Annex 29 Resolution A.1106(29). Available from: https://wwwcdn.imo.org/localresources/en/OurWork/Safety/Documents/AIS/Resolution%20A.1106(29).pdf

[ece311299-bib-0049] International Maritime Organisation . (2017). International Code for Ships Operating in Polar Waters (Polar Code). Available from: https://wwwcdn.imo.org/localresources/en/MediaCentre/HotTopics/Documents/POLAR%20CODE%20TEXT%20AS%20ADOPTED.pdf

[ece311299-bib-0050] International Maritime Organisation . (2023). Guidelines for the control and management of ships' biofouling to minimize the transfer of invasive aquatic species. Annex 17 Resolution MEPC.378(80). Available from: https://wwwcdn.imo.org/localresources/en/KnowledgeCentre/IndexofIMOResolutions/MEPCDocuments/MEPC.378(80).pdf

[ece311299-bib-0051] Jeschke, J. M. , Bacher, S. , Blackburn, T. M. , Dick, J. T. A. , Essl, F. , Evans, T. , Gaertner, M. , Hulme, P. E. , Kühn, I. , Mrugała, A. , Pergl, J. , Pyšek, P. , Rabitsch, W. , Ricciardi, A. , Richardson, D. M. , Sendek, A. , Vilà, M. , Winter, M. , & Kumschick, S. (2014). Defining the impact of non‐native species. Conservation Biology, 28, 1188–1194. 10.1111/cobi.12299 24779412 PMC4282110

[ece311299-bib-0052] Keller, R. P. , Drake, J. M. , Drew, M. B. , & Lodge, D. M. (2011). Linking environmental conditions and ship movements to estimate invasive species transport across the global shipping network. Diversity and Distributions, 17, 93–102. 10.1111/j.1472-4642.2010.00696.x

[ece311299-bib-0053] Kennicutt, M. C. , Bromwich, D. , Liggett, D. , Njåstad, B. , Peck, L. , Rintoul, S. R. , Ritz, C. , Siegert, M. J. , Aitken, A. , Brooks, C. M. , Cassano, J. , Chaturvedi, S. , Chen, D. , Dodds, K. , Golledge, N. R. , Le Bohec, C. , Leppe, M. , Murray, A. , Nath, P. C. , … Chown, S. L. (2019). Sustained Antarctic research: A 21st century imperative. One Earth, 1, 95–113. 10.1016/j.oneear.2019.08.014

[ece311299-bib-0054] Key, J. (2018). Tackling invasive non‐native species in the UK overseas territories: Pathway analyses. Available from: https://www.nonnativespecies.org/assets/Pathway_Analyses_Final_Report_March2018.pdf

[ece311299-bib-0055] Key, J. , & Moore, N. P. (2019). Tackling invasive non‐native species in the UK overseas territories. Island Invasives: Scaling up to Meet the Challenge, 62, 637. Available from: https://www.sprep.org/attachments/VirLib/Global/tackling‐invasive‐non‐native‐species‐uk.pdf

[ece311299-bib-0056] Lee, J. E. , & Chown, S. L. (2007). Mytilus on the move: Transport of an invasive bivalve to the Antarctic. Marine Ecology Progress Series, 339, 307–310. 10.3354/meps339307

[ece311299-bib-0057] Lee, J. E. , & Chown, S. L. (2009). Temporal development of hull‐fouling assemblages associated with an Antarctic supply vessel. Marine Ecology Progress Series, 386, 97–105. 10.3354/meps08074

[ece311299-bib-0058] Letschert, J. , Wolff, M. , Kluger, L. C. , Freudinger, C. , Ronquillo, J. , & Keith, I. (2021). Uncovered pathways: Modelling dispersal dynamics of ship‐mediated marine introduced species. Journal of Applied Ecology, 58, 620–631. 10.1111/1365-2664.13817

[ece311299-bib-0059] Lewis, P. N. , Hewitt, C. , Riddle, M. , & McMinn, A. (2003). Marine introductions in the Southern Ocean: An unrecognised hazard to biodiversity. Marine Pollution Bulletin, 46, 213–223. 10.1016/S0025-326X(02)00364-8 12586117

[ece311299-bib-0060] Lockwood, J. L. , Hoopes, M. F. , & Marchetti, M. P. (2013). Invasion Ecology (2nd edition). John Wiley & Sons.

[ece311299-bib-0061] López‐Farrán, Z. , Guillaumot, C. , Vargas‐Chacoff, L. , Paschke, K. , Dulière, V. , Danis, B. , Poulin, E. , Saucède, T. , Waters, J. , & Gérard, K. (2021). Is the southern crab Halicarcinus planatus (Fabricius, 1775) the next invader of Antarctica? Global Change Biology, 27, 3487–3504. 10.1111/gcb.15674 33964095

[ece311299-bib-0062] Marbuah, G. , Gren, I. M. , & McKie, B. (2014). Economics of harmful invasive species: A review. Diversity, 6, 500–523. 10.3390/d6030500

[ece311299-bib-0063] Marine Management Organisation . (2013). Spatial Trends in Shipping Activity. A report produced for the Marine Management Organisation (MMO), pp. 46. MMO Project No: 1042. Available from: https://repository.oceanbestpractices.org/handle/11329/1690

[ece311299-bib-0064] McCarthy, A. H. , Peck, L. S. , & Aldridge, D. C. (2022). Ship traffic connects Antarctica's fragile coasts to worldwide ecosystems. Proceedings of the National Academy of Sciences, 119, e2110303118. 10.1073/pnas.2110303118 PMC8784123 35012982

[ece311299-bib-0065] McCarthy, A. H. , Peck, L. S. , Hughes, K. A. , & Aldridge, D. C. (2019). Antarctica: The final frontier for marine biological invasions. Global Change Biology, 25, 2221–2241. 10.1111/gcb.14600 31016829 PMC6849521

[ece311299-bib-0066] McDonald, J. I. , Wellington, C. M. , Coupland, G. T. , Pedersen, D. , Kitchen, B. , Bridgwood, S. D. , Hewitt, M. , Duggan, R. , & Abdo, D. A. (2020). A united front against marine invaders: Developing a cost‐effective marine biosecurity surveillance partnership between government and industry. Journal of Applied Ecology, 57, 77–84. 10.1111/1365-2664.13557

[ece311299-bib-0067] McGeoch, M. A. , Genovesi, P. , Bellingham, P. J. , Costello, M. J. , McGrannachan, C. , & Sheppard, A. (2016). Prioritizing species, pathways, and sites to achieve conservation targets for biological invasion. Biological Invasions, 18, 299–314. 10.1007/s10530-015-1013-1

[ece311299-bib-0068] Molnar, J. L. , Gamboa, R. L. , Revenga, C. , & Spalding, M. D. (2008). Assessing the global threat of invasive species to marine biodiversity. Frontiers in Ecology and the Environment, 6, 485–492. 10.1890/070064

[ece311299-bib-0069] Moser, C. S. , Wier, T. P. , Grant, J. F. , First, M. R. , Tamburri, M. N. , Ruiz, G. M. , Miller, A. W. , & Drake, L. A. (2016). Quantifying the total wetted surface area of the world fleet: A first step in determining the potential extent of ships' biofouling. Biological Invasions, 18, 265–277. 10.1007/s10530-015-1007-z

[ece311299-bib-0070] Mrowicki, R. J. , & Brodie, J. (2023). The first record of a non‐native seaweed from South Georgia and confirmation of its establishment in The Falkland Islands: Ulva fenestrata Postels & Ruprecht. Polar Biology, 46, 489–496. 10.1007/s00300-023-03136-6

[ece311299-bib-0071] Navarro, J. M. , Cárdenas, L. , Ortiz, A. , Figueroa, A. , Morley, S. A. , Vargas‐Chacoff, L. , Leclerc, J. , & Détrée, C. (2024). Testing the physiological capacity of the mussel *Mytilus chilensis* to establish into the Southern Ocean. Science of the Total Environment, 921, 170941. 10.1016/j.scitotenv.2024.170941 38360303

[ece311299-bib-0072] Peck, L. S. , Morley, S. A. , Richard, J. , & Clark, M. S. (2014). Acclimation and thermal tolerance in Antarctic marine ectotherms. The Journal of Experimental Biology, 217, 16–22. 10.1242/jeb.089946 24353200

[ece311299-bib-0073] Peck, L. S. , Webb, K. E. , & Bailey, D. M. (2004). Extreme sensitivity of biological function to temperature in Antarctic marine species. Functional Ecology, 18, 625–630. 10.1111/j.0269-8463.2004.00903.x

[ece311299-bib-0074] Queirós, J. P. , Xavier, J. C. , Abreu, J. , Collins, M. A. , Belchier, M. , & Hollyman, P. R. (2024). What inhabits the South Sandwich Islands deep‐sea? Biodiversity and biogeography of bathyal communities using predators as biological samplers. Deep Sea Research Part I: Oceanographic Research Papers, 205, 104260. 10.1016/j.dsr.2024.104260

[ece311299-bib-0075] Ricciardi, A. , & Cohen, J. (2007). The invasiveness of an introduced species does not predict its impact. Biological Invasions, 9, 309–315. 10.1007/s10530-006-9034-4

[ece311299-bib-0076] Roy, H. E. , Peyton, J. M. , Pescott, O. L. , Rorke, S. L. , Ecology, C. , Gifford, C. , Adriaens, T. , Cottier‐cook, E. , Dawson, W. , Frohlich, D. , Malumphy, C. , Martinou, A. F. , Minchin, D. , Rabitsch, W. , Rorke, S. L. , Tricarico, E. , Turvey, K. M. A. , & Winfield, I. (2019). Prioritising invasive non‐native species through horizon scanning on the UK overseas territories. 10.13140/RG.2.2.18951.34726

[ece311299-bib-0077] Saebi, M. , Xu, J. , Grey, E. K. , Lodge, D. M. , Corbett, J. J. , & Chawla, N. (2020). Higher‐order patterns of aquatic species spread through the global shipping network. PLoS One, 15, 1–24. 10.1371/journal.pone.0220353 PMC7394518 32735579

[ece311299-bib-0078] Seebens, H. , Gastner, M. T. , & Blasius, B. (2013). The risk of marine bioinvasion caused by global shipping. Ecology Letters, 16, 782–790. 10.1111/ele.12111 23611311

[ece311299-bib-0079] Simberloff, D. (2011). Correspondence: Non‐natives: 141 scientists object. Nature, 475, 36. 10.1038/475036a 21734689

[ece311299-bib-0080] Spalding, M. D. , Fox, H. E. , Allen, G. R. , Davidson, N. , Ferdaña, Z. A. , Finlayson, M. , Halpern, B. S. , Jorge, M. A. , Lombana, A. , Lourie, S. A. , Martin, K. D. , Mcmanus, E. , Molnar, J. , Recchia, C. A. , & Robertson, J. (2007). Marine ecoregions of the world: A bioregionalization of coastal and shelf areas. Bioscience, 57, 573–583. 10.1641/B570707

[ece311299-bib-0081] Stammerjohn, S. , Massom, R. , Rind, D. , & Martinson, D. (2012). Regions of rapid sea ice change: An inter‐hemispheric seasonal comparison. Geophysical Research Letters, 39, 1–8. 10.1029/2012GL050874

[ece311299-bib-0082] Sylvester, F. , Kalaci, O. , Leung, B. , Lacoursière‐Roussel, A. , Murray, C. C. , Choi, F. M. , Bravo, M. A. , Therriault, T. W. , & Macisaac, H. J. (2011). Hull fouling as an invasion vector: Can simple models explain a complex problem? Journal of Applied Ecology, 48, 415–423. 10.1111/j.1365-2664.2011.01957.x

[ece311299-bib-0042] UNEP‐WCMC . (2021). Protected area profile for South Georgia and South Sandwich Islands marine protected area [WWW document]. World Database on Protected Areas. https://www.protectedplanet.net/555547601

[ece311299-bib-0083] Varnham, K. (2006). Non‐native species in UK overseas territories: A review, JNCC Report No. 372, JNCC, Peterborough. Available from: https://data.jncc.gov.uk/data/bdb47e73‐aa8b‐4958‐8658‐b2e7f758e5bb/JNCC‐Report‐372‐FINAL‐WEB.pdf

[ece311299-bib-0084] Williams, S. L. , Davidson, I. C. , Pasari, J. R. , Ashton, G. V. , Carlton, J. T. , Crafton, R. E. , Fontana, R. E. , Grosholz, E. D. , Miller, A. W. , Ruiz, G. M. , & Zabin, C. J. (2013). Managing multiple vectors for marine invasions in an increasingly connected world. Bioscience, 63, 952–966. 10.1525/bio.2013.63.12.8

[ece311299-bib-0085] Zabin, C. J. , Ashton, G. V. , Brown, C. W. , Davidson, I. C. , Sytsma, M. D. , & Ruiz, G. M. (2014). Small boats provide connectivity for nonindigenous marine species between a highly invaded international port and nearby coastal harbors. Management of Biological Invasions, 5, 97–112. 10.3391/mbi.2014.5.2.03

[ece311299-bib-0086] Zabin, C. , Davidson, I. , Holzer, K. , Smith, G. , Ashton, G. , Tamburri, M. , & Ruiz, G. (2018). How will vessels be inspected to meet emerging biofouling regulations for the prevention of marine invasions? Management of Biological Invasions, 9(3), 195–208. 10.3391/mbi.2018.9.3.03

