# Supplementary material for: Identifying marine invasion threats and management priorities through introduction pathway analysis in a remote sub‐Antarctic ecosystem
Source: Ecol Evol. 2024 Apr 23;14(4):e11299. doi: 10.1002/ece3.11299 (PMC11036081; doi:10.1002/ece3.11299)

# APPENDICES

Appendix 1. Summary of vessel movement and specification characteristics for vessel mean and total values assessed using AIS tracking data over 2 years, July 2017-2019. All data for vessels moving within the inshore waters of South Georgia and the South Sandwich Islands, for the 10 busiest SGSSI ports / anchorages.


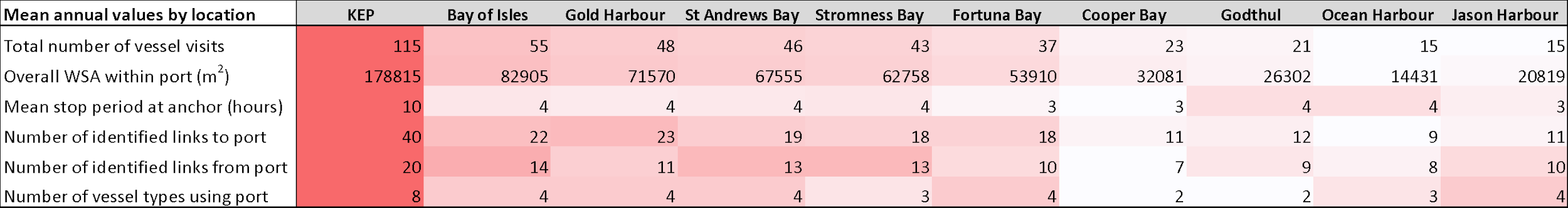

Supplement: Supplementary file 1 — Appendix S1. [file ECE3-14-e11299-s001.docx]
